# Healthy together Victoria and childhood obesity study: effects of a large scale, community-based cluster randomised trial of a systems thinking approach for the prevention of childhood obesity among secondary school students 2014–2016

**DOI:** 10.1186/s12889-024-17906-2

**Published:** 2024-02-02

**Authors:** Claudia Strugnell, Liliana Orellana, Nicholas Crooks, Mary Malakellis, Bridget Morrissey, Claire Rennie, Joshua Hayward, Jo Bliss, Boyd Swinburn, Cadeyrn J. Gaskin, Steven Allender

**Affiliations:** 1 Faculty of Health, Global Centre for Prevention Health and Nutrition (GLOBE), Institute for Health Transformation, Deakin University, Geelong, Australia; 2 Faculty of Health, Biostatistics Unit, Deakin University, Geelong, Australia; 3 School of Population Health, University of Auckland, Auckland, New Zealand; 4 Institute for Physical Activity and Nutrition, Deakin University Waterfront Campus, Geelong, Victoria 3220 Australia

**Keywords:** Exercise, Health promotion, Overweight, Obesity, Sugar-sweetened beverages, Victoria, Waist circumference

## Abstract

**Background:**

Healthy Together Victoria (HTV) was a Victorian Government initiative that sought to reduce the prevalence of overweight and obesity through targeting chronic disease risk factors including physical activity, poor diet quality, smoking, and harmful alcohol use. The intervention involved a boosted workforce of > 170 local-level staff in 12 communities; employed to deliver system activation around health and wellbeing for individuals, families and communities. A cluster randomised trial (CRT) of a systems thinking approach to obesity prevention was embedded within HTV. We present the two-year changes in overweight and obesity and associated behaviours among secondary school students across Victoria, Australia.

**Methods:**

Twenty-three geographically bounded areas were randomised to intervention (12 communities) or comparison (11 communities). Randomly selected secondary schools within each community were invited to participate in the trial in 2014 and 2016. Students in Grade 8 (aged approximately 13–15 years) and Grade 10 (aged approximately 15–16 years) at participating schools were recruited using an opt-out approach across July–September 2014 and 2016. Primary outcomes were body mass index (BMI) and waist circumference. Secondary outcomes were physical activity, sedentary behaviour, diet quality, health-related quality of life, and depressive symptoms. Linear mixed models were fit to estimate the intervention effect adjusting for child/school characteristics.

**Results:**

There were 4242 intervention children and 2999 control children in the final analysis. For boys, the two-year change showed improvement in intervention versus control for waist circumference (difference in change: − 2.5 cm; 95% confidence interval [CI]: − 4.6, − 0.5) and consumption of sugar-sweetened beverages per day (< 1 serve: 8.5 percentage points; 95% CI: 0.6, 16.5). For girls, there were no statistically significant differences between conditions.

**Conclusions:**

HTV seemed to produce favourable changes in waist circumference and sugar-sweetened beverage consumption for boys, however, no effect on BMI was observed. Although the HTV intervention was cut short, and the period between data collection points was relatively short, the changes observed in HTV contribute to the growing evidence of whole-of-community interventions targeting childhood obesity.

**Trial registration:**

This trial is unregistered. The intervention itself was a policy setting delivered by government and our role was the collection of data to evaluate the effect of this natural experiment. That is, this study was not a trial from the classical point of view and we were not responsible for the intervention.

**Supplementary Information:**

The online version contains supplementary material available at 10.1186/s12889-024-17906-2.

## Background

Prevention of childhood obesity is of critical public health importance [1], not only to avert persistence into adulthood [2], but to reduce the associated acute and chronic conditions evident during childhood [3]. Globally, childhood overweight and obesity have risen dramatically since the mid-1970s, with over 300 million children in 2016 affected [4]. To date, no country has reversed their obesity epidemic, with the systemic and institutional drivers (food and agriculture, transportation, urban design, and land use) largely continuing unrestricted [5]. In Australia, 24.9% of children aged 5–17 years were with overweight or obesity in 2017–18 [6].

Evidence from recent meta-analyses has shown that interventions for preventing obesity in school students are effective for reducing body mass index [7, 8]. An update of a Cochrane systematic review and meta-analysis, for example, found that school-based obesity prevention interventions had a small but beneficial effect on body mass index (standardised mean difference (SMD) = − 0·03; 95% confidence interval [CI]: − 0·06, − 0·01; trials = 93; participants = 31,443; moderate certainty evidence) [7]. No effects on body mass index (BMI) were found for after-school program interventions (SMD = -0·09; 95% CI: − 0·22, − 0·04; trials = 12; participants = 5066; very low-certainty evidence), community-based interventions (SMD = -0·04; 95% CI: − 0·11, 0·04; trials = 21; participants = 3292; low certainty evidence), or home-based interventions (SMD = 0·01; 95%: CI -0·07, 0·09; trials = 13; participants = 2400; low certainty evidence) [7]. Promising strategies for reducing BMI are multi-component interventions that often engage key stakeholders (e.g., teachers, parents), include digital interventions, and modify environments [8].

Systems thinking (or systems science) is an established scientific field that is increasingly being applied to the prevention of non-communicable diseases and obesity [9, 10]. Systems thinking begins with the premise that largely intractable problems, like obesity, are complex, and interventions should be designed in a way that recognises this inherent complexity [11]. Approaches using methods from systems thinking for obesity prevention have emerged over the last two decades, with key international examples including the *B’More Healthy Communities for Kids* in Baltimore (2013–2016), United States [12], and the *Whole Systems Approach to Obesity* in England [13]. Examples of systems thinking approaches in Australia include *Romp & Chomp* in Geelong (2004–2008) [14, 15] and *Whole of Systems Trial of Prevention Strategies (WHOSTOPS) for childhood obesity* (2015–2021) [16]. The effects of these interventions on BMI z-scores (BMIz) have tended to range from − 0.04 (*Romp & Chomp* [14]) to − 0.10 (first-year results from *Shape Up Sommerville* in the United States [17]). Favourable effects on behaviours associated with overweight and obesity (e.g., less screen time [14], more healthy eating [14]) have also been demonstrated. Much of this work has been conducted with primary school-aged children with comparatively fewer studies including adolescents.

At the forefront of these efforts was the Healthy Together Victoria (HTV) initiative in Victoria, Australia, which ran from 2012 to 2016. HTV involved the use of systems thinking to address rising rates of obesity and preventing obesity-related chronic conditions through improvements in associated behavioural determinants (physical inactivity, poor diet quality, harmful alcohol use, and smoking) [18, 19]. The initiative involved a boosted workforce of approximately 170 staff in 12 communities using systems thinking to support and deliver *systems activation* (i.e., initiating actions on the systems that influence the health and well-being of individuals, families, and communities) for healthier schools, early childhood settings, workplaces, and communities through various means where Victorians “live, learn, work and play” [19].

The evaluation included a cluster randomised trial (CRT) design to investigate outcomes for secondary school students. In this paper, we present the effects of the HTV initiative over 2 years (2014–2016) on overweight and obesity and associated behaviours (physical activity, sedentary behaviour, and diet quality) among secondary school students aged 13–14 and 15–16 years. Although the initiative started in 2012, the first data collection point was in 2014 (so does not represent a true baseline) when many schools had not yet heard of HTV. Planning occurred in 2012 with implementation beginning in 2013.

## Methods

The Victorian Department of Health and Local Municipal Associations in Victoria designed and implemented the HTV initiative, with funding from the Commonwealth Government of Australia and the State Government of Victoria [19, 20]. The CRT reported here – the HTV and Childhood Obesity study – was conducted independently of government and involved the collection and analysis of anthropometric, behavioural, health-related quality of life, and depressive symptom data from secondary school students. The government’s HTV initiative and the research method are described in the study’s protocol [20]. A summary is provided here.

### Design

The HTV and Child Obesity study was a CRT involved 23 communities within a repeat-cross-sectional evaluation design [20]. The Victorian Department of Health selected the 23 communities that would participate in the trial, organised them in 11 strata (one with 3 communities) based on socio-demographic indices (Socioeconomic Index for Areas [SEIFA] [21]) and prevalence of unhealthy weight in adults. Adult BMI data was used for stratification because the HTV initiative targeted all ages. Communities were randomly allocated to intervention condition (*n* = 12) or comparison (*n* = 11).

### School and participant selection

A list of randomly selected schools was produced for each of the 23 participating communities. Written invitations and follow-up phone calls, emails, and/or in-person visits were held with school principals of the first three schools in the list to ascertain school participation. If all three secondary schools declined participation, then the next three schools were invited to participate until at-least one school per community participated, all schools within the community had been invited, and/or the data collection window expired. In 2016, all participating schools in 2014 were re-invited to participate, with a booster random sample of 3 schools for each community. Within each school, all Grade 8 (typically aged 13–14 years old), and Grade 10 (typically aged 15–16 years old) students were invited to participate via a 5–10 minute in-school presentation from the research team and an item about the study placed in the school newsletter. Each student received a written plain language statement and opt-out form. All students were enrolled in the study unless they returned an opt-out form signed by their parent/guardian or verbally opted out on the day. On the day of testing, students were re-informed that participation was voluntary and that they could participate as much as they like (e.g., only have their height measured, only complete the questionnaire) or opt-out at any stage. Consent was implied if children did not opt out. Data collection occurred across July–September 2014 and 2016.

### Sample size

The original power calculation was based on a sample of students from both primary and secondary schools, opt-in consent, and a four-year intervention (2012 to 2014) [20]. As previously published, (19) in order to detect a BMIz score change between intervention and control communities of 0.18 with a power of 0.8 and alpha of 0.05. A sample size of ≥25 children in each of the 4 -year levels (Grade 4, Grade 6, Grade 8 and Grade 10) is to be recruited in each of the 12 intervention and 12 control clusters (12*25*4 = 1200 intervention) and (12*25*4 = 1200 control). We substantially exceeded the required sample size due to the approval of an opt-out approach and have met the minimum 1200 intervention and 1200 control to examine the data separately for Grade 8 and Grade 10 [19]. Due to extremely low recruitment rates using an opt-in approach in 2013, we obtained ethics approval to use an opt-out approach in 2014 and 2016. We present here the analysis of the outcomes collected using opt-out approach for secondary school students in the years 2014 and 2016. No a priori power calculation was undertaken for this analysis.

### Intervention

HTV sought to take a whole of population approach to addressing rising obesity rates and preventing obesity-related chronic conditions, and involved the implementation of multiple strategies, policies, and initiatives at both the state and local levels [18, 19]. The HTV intervention targeted early childhood services, primary and secondary schools, and medium to large workplaces. Actions taken included: political leadership for health promotion; providing quality improvement frameworks for early childhood services, schools, and workplaces; supporting policy development/implementation and targeted strategies within settings (e.g. the multicomponent health and wellbeing Achievement Program within early childhood services, schools, and workplaces [22]); working with food growers, producers, and sellers to increase access to healthy food, especially for disadvantaged populations; working with urban planning to support active transport; working with sport and recreation centres to increase healthy food access; building leadership for prevention; and social marketing of health promotion messaging [18]. Due to the broad scope and the adaptive nature of HTV, providing detailed information is beyond the scope of this paper. A brief video on HTV is available at https://www.youtube.com/watch?v=pZU8MYGqm2s and a summary of one community’s experience (Mildura) has been published [23].

### Demographics

Students reported date of birth, gender, residential postcode or town/suburb, Aboriginal and/or Torres Strait Islander background, language spoken at home, country of birth, and ancestry. The Index of Community Socio-Educational Advantage (ICSEA) was used to examine socioeconomic background at the school-level [24]. ICSEA is a measure of the average educational advantage of a school’s student population compared with other schools based on child characteristics (parent/guardian occupation and education) and school factors (geographic location and proportion of Indigenous students) [24]. ICSEA has a median value of 1000, with higher values indicative of greater socioeconomic advantage. School-level ICSEA scores were obtained from the Australian Curriculum, Assessment and Reporting Authority’s *My School* website [25].

### Primary outcomes

#### Body mass index

Height and weight were measured twice without shoes and while wearing light clothing to the nearest 0.1 cm for height (Charder HM-200P Portstad, Charder Electronic Co Ltd., Taichung City, Taiwan) and 0.1 kg for weight (A&D Precision Scale UC-321; A7D Medical, San Jose, CA). A third measurement was taken if there was a discrepancy of ≥0.5 cm for height or > 0.5 kg for weight in the initial 2 measurements. Height and weight measurements were averaged for analysis. Healthy weight (including underweight), overweight and obesity were defined using the World Health Organization’s age- and sex-specific BMI growth reference [26].

#### Waist circumference

Waist circumference was taken over light clothing using the cross-over technique at the midway point between the lower costal border (10th rib) and the top of the iliac crest, in the mid-axillary line, perpendicular to the long axis of the trunk [27] using a steel tape measure (Lufkin W606PM) to the nearest 0.1 cm. Two measurements were taken, with a third measurement made if there was a discrepancy of > 0.5 cm between the first two measurements. The measurements were then averaged for analysis. Abdominal obesity was classified when mean waist circumference was greater ≥90th percentile [28] according to the International Diabetes Federation using the British age- and sex-specific growth reference [29].

#### Secondary outcomes

A questionnaire was administered during class time (approximately 20–30 minutes), which captured data on physical activity, sedentary behaviour, diet quality, health-related quality of life, and depressive symptoms.

#### Physical activity and sedentary behaviour

Physical activity and sedentary behaviour were assessed with 11 items from the School Health Action, Planning and Evaluation System questionnaire [30] and three items from the Canadian Core Indicators and Measures of Youth Health – Physical Activity & Sedentary Behaviour Module questionnaire. These question items examined self-reported time spent in moderate-to-vigorous physical activity (MVPA), sedentary behaviour, and mode of active transport to and from school [30]. Data on time spent in MVPA and sedentary behaviour were used to determine adherence to the 2014 Australian physical activity and sedentary behaviour guidelines of ≥60 minutes/day of MVPA and ≤ 2 hrs/day of sedentary recreational screen time [31, 32].

#### Diet quality

A modified nine-item version of a basic dietary questionnaire [33] was used to examine usual intake of core foods and beverages (e.g. fruit, vegetables, and unsweetened dairy products) and non-core foods and beverages (e.g. takeaway foods and sugar-sweetened beverages). Diet data were used to examine daily adherence to the 2013 Australian Dietary Guidelines of two serves of fruit for those aged 9 to 18 years, five serves of vegetables for girls aged 9 to 18, and 5.5 serves of vegetables for boys aged 12 to 18 years [34].

#### Health-related quality of life

The Paediatric Quality of Life Inventory 4.0 (PedsQL™ 4.0) Generic Core Scale [35] was used as a measure of health-related quality of life. PedsQL is a 23-item questionnaire that examines four domains: physical functioning (8 items), emotional functioning (5 items), school functioning (5 items), and social functioning (5 items). The domain scores were summed to provide an overall global health-related quality of life score and summary scores for physical health (generated from items in the physical functioning domain) and psychosocial health (generated from items in the emotional, social, and school functioning domains).

#### Depressive symptoms

The Short Mood and Feelings Questionnaire (SMFQ) [36, 37] was used to measure symptoms of depression. With the SMFQ, responds complete a checklist of 13 items about how they have been feeling or acting during the past 2 weeks (e.g., “I feel miserable or unhappy”) using a three-point scale: *not true* (scored as 0), *sometimes* (scored as 1), and *true* (scored as 2). Scores for each of the items were summed, with higher scores indicative of greater depressive symptoms (range 0–26). There are no prescribed cut points for the SMFQ, but initial research showed that scores of 8 or more predicted depression with 60% sensitivity and 85% specificity [36, 37].

### Data analysis

We conducted a repeat cross-sectional analysis on data collected from 35 secondary schools. All analyses were conducted using an intention-to-treat approach (i.e., independently of whether the intervention communities had received any level of intervention and whether the comparison communities had implemented other strategies to improve healthy behaviours or prevent obesity). We estimated the change between 2014 and 2016 on continuous outcomes (BMIz, waist circumference, abdominal obesity, health-related quality of life, and depressive symptoms) for each group and the difference in change between groups using linear mixed models. The same estimates for binary variables (overweight and obese; physical activity, sedentary behaviour, and dietary behaviours; and potential depressive disorder) were obtained using a generalised estimating equations (GEE) approach (logit link and binomial distribution). All models included fixed effects: group (intervention/comparison), wave (2014, 2016), interaction group × wave; and two school level factors, ICSEA (≤999, ≥1000), and location of the school (major cities, inner regional, outer regional). The school level factors were incorporated to adjust for imbalances in the socioeconomic level of schools participating at different waves. Clustering induced by school was accounted for by including school as random effect in linear mixed models and considering a working covariance matrix with compound symmetry in GEE models. The same models were fitted for each gender separately. No adjustment for multiplicity of outcomes was considered. All analyses were performed using SAS (version 9.4).

## Results

Of the secondary schools invited in each study year, 23/146 (15%) participated in 2014 and 28/136 (21%) participated in 2016. Twelve (*n* = 12) schools participated in both 2014 and 2018 (52% of the original sample). Student participation rates were high among both intervention 2021/2344 (86.2%) and control 1273/ 1503 (84.7%) schools in 2014 and intervention 2170/2450 (88.6%) and control (1754/1921) (91.3%) schools in 2016 (Supplementary Table 1). Demographic characteristics of the students are provided in Table 1 and Supplementary Tables 2, and 3. Their average age was 15 years and approximately three quarters spoke predominantly English at home.

**Table 1:** Demographic characteristics and anthropomorphic, behavioural, quality of life, and depressive symptom outcomes by wave and trial condition for secondary school students

	Intervention				Comparison				Change between 2016 and 2014				Difference in change	
	2014		2016		2014		2016		Intervention		Comparison		Intervention vs Comparison	
	*N*	Estimate (95% CI)	*N*	Estimate (95% CI)	*N*	Estimate (95% CI)	*N*	Estimate (95% CI)	Estimate (95% CI)	*P*	Estimate (95% CI)	*P*	Estimate (95% CI)	*P*
**Demographic Characteristics**														
Age (years)	2011	15.0 (14.8, 15.2)	2231	15.0 (14.8, 15.2)	1270	15.0 (14.8, 15.2)	1729	14.9 (14.7, 15.2)	0.0 (−0.1, 0.1)	0.935	0.0 (− 0.1, 0.1)	0.460	0.0 (− 0.2, 0.1)	0.639
English spoken at home (%)	1985	76.7 (64.8, 88.6)	2206	77.1 (65.8, 88.4)	1244	75.6 (65.3, 85.9)	1673	76.4 (65.6, 87.2)	0.8 (−3.9, 5.5)	0.727	0.4 (−1.7, 2.5)	0.734	−0.5 (−5.6, 4.7)	0.856
**Anthropometrics**														
BMIz (WHO)	1741	0.55 (0.43, 0.66)	1940	0.56 (0.46, 0.67)	1139	0.46 (0.33, 0.59)	1394	0.54 (0.42, 0.67)	0.01 (−0.1, 0.13)	0.811	0.08 (−0.03, 0.2)	0.143	−0.07 (− 0.23, 0.09)	0.382
Overweight and obese (%)	1741	34.4 (30.4, 38.4)	1940	33.8 (29.7, 38)	1139	29.6 (25.9, 33.2)	1394	32.5 (28.1, 36.9)	−0.6 (−4.2, 3)	0.743	2.9 (−0.7, 6.5)	0.116	−3.5 (−8.8, 1.8)	0.196
Waist circumference (cm)	1782	75.7 (74.4, 77)	1913	75.2 (74, 76.4)	1152	74.4 (73, 75.8)	1391	75.9 (74.5, 77.3)	−0.5 (−1.5, 0.6)	0.361	1.5 (0.5, 2.6)	0.005	−2.0 (−3.5, −0.5)	0.008
Abdominal obesity (%)	1782	16.5 (12.9, 20.1)	1913	17.8 (13.5, 22.1)	1152	13 (9.4, 16.7)	1391	16 (12.5, 19.5)	1.3 (−1.6, 4.3)	0.383	3 (−1.1, 7.1)	0.153	−1.7 (−7, 3.6)	0.537
**Behaviour**														
Met physical activity guideline (5 days) (%)	1988	22.8 (20.1, 25.4)	2217	25.6 (23.3, 28)	1255	23.1 (19.7, 26.4)	1682	24.7 (20.7, 28.7)	2.8 (0.1, 5.5)	0.040	1.6 (−3.9, 7.2)	0.557	1.2 (−4.7, 7.1)	0.692
Met sedentary guideline (5 days) (%)	1982	52.9 (47.6, 58.1)	2105	55.8 (51.6, 59.9)	1253	47.4 (43.4, 51.5)	1599	53 (49.5, 56.6)	2.9 (−1.6, 7.4)	0.207	5.6 (2.6, 8.6)	< 0.001	−2.7 (−7.9, 2.5)	0.312
Active transport to or from school (%)	1848	34.5 (27.6, 41.4)	2217	34.4 (27.9, 40.9)	1174	30.9 (23.9, 37.8)	1685	33.7 (28.5, 38.9)	−0.1 (−2.5, 2.3)	0.963	2.8 (−2.5, 8.2)	0.297	−2.9 (−8.5, 2.8)	0.316
Met fruit guideline (%)	1983	67.4 (61.9, 72.8)	2148	63.6 (59, 68.2)	1244	70.8 (66.3, 75.3)	1640	67.5 (64, 71.1)	−3.7 (−7.5, 0)	0.052	−3.3 (−6.9, 0.4)	0.079	−0.5 (−5.6, 4.6)	0.849
Met vegetable guideline (%)	1981	6.4 (4.9, 7.9)	2211	9.8 (8.1, 11.5)	1238	5.4 (4.1, 6.8)	1679	10.1 (8.5, 11.6)	3.4 (1.6, 5.3)	< 0.001	4.6 (3.1, 6.2)	< 0.001	−1.2 (−3.5, 1.1)	0.318
Dairy (≥3 serves/day) (%)	1974	72.6 (69.7, 75.5)	2214	64.4 (61.6, 67.1)	1251	70.1 (67.2, 73)	1681	64.9 (60.8, 69)	−8.3 (−11.6, −5)	< 0.001	−5.2 (−9.6, −0.8)	0.022	−3.1 (− 8.5, 2.3)	0.266
Sugar-sweetened beverages (< 1/day) (%)	1978	25.4 (22.2, 28.6)	2214	25.1 (21.3, 28.8)	1253	27.3 (23.9, 30.6)	1676	21.6 (18.4, 24.8)	−0.3 (−3.3, 2.6)	0.828	−5.7 (−9.1, −2.2)	0.001	5.3 (0.7, 9.9)	0.023
Packaged snacks (< 1/day) (%)	1967	54.3 (50.7, 57.9)	1950	52.1 (48.3, 56)	1246	52.6 (47.8, 57.4)	1550	53 (48.5, 57.4)	−2.2 (−6.9, 2.6)	0.376	0.4 (−4.7, 5.5)	0.885	−2.5 (−9.4, 4.3)	0.467
Takeaway (≤1/week) (%)	1980	17.2 (14.4, 20)	2217	15.2 (12.1, 18.4)	1244	15.5 (12.4, 18.6)	1684	15.4 (11.8, 19.1)	−2 (−4.6, 0.6)	0.135	−0.1 (−2.5, 2.4)	0.959	−1.9 (−5.6, 1.7)	0.302
**Health-Related Quality of Life**														
Global score	1976	84.9 (83.8, 86.1)	2171	83.1 (82.2, 84.1)	1256	84 (82.8, 85.3)	1651	83.6 (82.4, 84.7)	−1.78 (−2.95, −0.61)	0.003	−0.46 (− 1.65, 0.73)	0.447	− 1.32 (− 2.97, 0.33)	0.117
Physical functioning	1980	74.9 (73.6, 76.2)	2196	72.1 (71, 73.3)	1257	73.2 (71.8, 74.6)	1670	71.9 (70.6, 73.3)	−2.75 (−4.1, −1.4)	< 0.001	−1.27 (− 2.64, 0.1)	0.070	− 1.48 (−3.39, 0.43)	0.128
Psychosocial functioning	1976	78.4 (77.3, 79.5)	2172	76 (75, 77)	1258	76.9 (75.7, 78.1)	1654	76 (74.8, 77.1)	−2.35 (−3.51, −1.2)	< 0.001	−0.95 (− 2.12, 0.23)	0.115	− 1.41 (− 3.04, 0.23)	0.092
**Depressive Symptoms**														
Depressive symptomatology	1917	4.5 (4.1, 4.9)	2071	5.4 (5, 5.7)	1220	5.1 (4.6, 5.6)	1579	5.7 (5.3, 6.2)	0.9 (0.4, 1.3)	0.001	0.6 (0.2, 1.1)	0.011	0.2 (−0.5, 0.9)	0.544
Potential depressive disorder (%)	1917	18.1 (14.6, 21.5)	2071	23.8 (21.2, 26.4)	1220	23.1 (19.7, 26.5)	1579	25.7 (23.6, 27.7)	5.7 (2.6, 8.8)	< 0.001	2.6 (0.2, 4.9)	0.034	3.2 (−0.6, 7)	0.101

In the overall analyses (boys and girls), between 2014 and 2016, students’ average waist circumference significantly increased in the comparison condition (+ 1.5 cm; 95% CI: 0.5, 2.6) but there was no significant change in the intervention condition (− 0.5 cm; 95% CI: − 1.5, 0.6) with a significant difference between groups (− 2.0 cm; 95% CI: − 3.5, − 0.5) (Table 1). There were no other significant differences between the two conditions in anthropometric measurements. With respect to behaviour, the percentage of students with low consumption of sugar sweetened beverages (< 1 sugar-sweetened beverage per day) significantly decreased in the comparison group (− 5.7 percentage points; 95% CI: 0.7, 9.9) while remaining constant in the intervention condition (− 0.3 percentage points; 95% CI: − 3.3, 2.6) with significant effect in favour of the intervention of (+ 5.3 percentage points; 95% CI: 0.7, 9.9). There were no other significant differences in behaviour between the two conditions. Nor were any differences apparent for health-related quality of life or depressive symptoms.

The results for the analyses of the data from boys only were similar to the overall results (Supplementary Table 2). Changes in waist circumference (− 2.5 cm; 95% CI: − 4.6, − 0.5) and sugar-sweetened beverage consumption of < 1 drink per day (8.5 percentage points; 95% CI: 0.6, 16.5) favoured boys in the intervention condition. There were no other statistically significant results for boys. For girls, no statistically significant differences between the two conditions were found (Supplementary Table 3).

## Discussion

HTV was one of the first attempts globally to implement a whole of community systems approach to obesity prevention and our findings suggest that it had a favourable impact on anthropometric outcomes and obesogenic behaviours for secondary school-aged boys within intervention communities. We found significant intervention effects for one of the primary outcomes (waist circumference) and one of the secondary outcomes (sugar-sweetened beverage consumption), both favouring boys in the intervention condition. The favourable change in waist circumference among boys of − 2.5 cm is similar to the change of − 3.1 cm found among children aged 4 to 12 years in the longitudinal *Be Active Eat Well* (2003–2006) community-based intervention in the Victorian town of Colac (Australia) [38]. Our findings show that favourable changes in waist circumference are also achievable among secondary school-aged boys in this repeat cross-sectional analyses.

A significant reduction in BMIz did not accompany the significant decrease in waist circumference. Even so, the changes in BMIz for the whole sample (− 0.07), and for boys (− 0.04), were in the anticipated direction and were of similar magnitude to the reductions observed with previous whole-of-community interventions, such as *Romp & Chomp* (− 0.04) [14], *Be Active Eat Well* (− 0.09) [39], and *Shape Up Sommerville* (− 0.10) [17]. The changes in BMIz observed in our study were also larger than in the effect found for diet combined with physical activity interventions in a recent meta-analysis (mean difference BMIz = 0.01, 95%CI: − 0.05, 0.07; 6 RCTs; *n* = 16,543) [40]. Our pattern of findings (significant waist circumference, nonsignificant BMIz) replicates those of a recent study on the association between sugar-sweetened beverage consumption and body composition in adolescents [41]. More work is required to understand why statistical significance might be achieved for waist circumference without the change in BMIz approaching significance.

There was an unexpected gender-specific effects for waist circumference and sugar-sweetened beverage consumption, which were favourable and statistically significant for intervention boys, but not for intervention girls. Although the purpose of the study was not to investigate gender-based differences, we noted that there were sizable differences between boys and girls in sugar-sweetened beverage consumption, abdominal obesity and meeting physical activity guidelines, each favouring boys. These observations are inconsistent with other Australian evidence that there are no differences between girls and boys in sugar-sweetened beverage consumption [42] or that boys’ consumption is higher [43]. Perhaps the boys in our sample happened to be more health conscious than the girls and were more receptive to messaging about sugar-sweetened beverage consumption.

### Strengths of this study

Strengths of this study include the embedding of the CRT within a natural experiment, scale, opt-out approach to the recruitment of adolescents, and measurement of waist circumference. Undertaking a CRT is relatively rare in evaluating systems thinking approaches for obesity prevention (WHOSTOPS is another example [16]), with non-randomised controlled trials, mixed methods evaluations, and case studies more common in the field [9]. The method is also rarely used in natural experiments like HTV and enabled the evaluation, in real time, of a government initiative. The trial evaluation was large in scale, with approximately 4000 adolescents from 23 communities participating at each of the two time points. The opt-out recruitment approach central to achieving high adolescent participation rates in 2014 (85.5%) and 2016 (90%) and exceeding the sample-size calculations of 1200 intervention and 1200 control; enabling analyses by secondary school only. These high participation rates compare favourably with student participation rates of 30–60% when opt-in procedures are used [44]. The use of waist circumference, together with BMIz, as a primary outcome recognises its strong correlation with abdominal subcutaneous adipose tissue, in particular, and visceral adipose tissue [45].

### Limitations of this study

First, the very low student participation rates among adolescents at baseline constrained our ability to collect sufficient data in 2013 (390/3789 invited adolescents, 11%). Traditional (i.e., opt-in) data collection approaches, like we attempted to use in 2013, are problematic for this type of research, because they are known to introduce participant bias [46]. Therefore, we support mandatory monitoring of height and weight (e.g., as part of government child monitoring programs) to enable the effectiveness of obesity prevention initiatives and policies to be accurately assessed [47, 48]. Our use of 2014 data in the present analysis does not represent a true baseline. Given the scale of what was being attempted in HTV, however, we observed that momentum for HTV was just emerging in 2014 within intervention schools. Second, the intervention was terminated suddenly – operating for only 4 of the 6 years planned – due to an abrupt cessation of the National Partnership Agreement on Preventative Health that had provided funding for HTV. If the trial had continued, the momentum of the intervention may have produced more pronounced changes in the primary and or secondary outcomes. Third, the first data collection point for this analysis (2014) occurred 1 year after implementation had begun. Although many of the schools had not heard of HTV at the time these data were collected, it is possible that HTV affected the 2014 data. Fourth, data collection on risks for obesity (e.g., physical activity, sedentary behaviour, and diet quality) relied upon self-report measures. These types of instruments are subject to various measurement errors (e.g., respondent bias, respondent memory lapses [49], incorrect estimation of portion sizes, and complexity in estimating mixed dishes [50]) and the results should be considered in this light. Fifth, obesity prevention initiatives may have been initiated or ongoing in comparison communities at the time of the trial. Further, there is a possibility that HTV-related activities may have spread beyond the intervention communities. If they were in place, obesity prevention initiatives in control communities could have attenuated the observed effects of HTV. Sixth, we have not controlled for multiple comparisons in our analysis and, therefore, the possibility of false positives cannot be ruled out. Finally, baseline BMIz scores and prevalence of overweight/obesity were lower in children from comparison communities which may partially explain the positive change in both outcomes observed in the comparison communities.

## Conclusions

HTV appeared to have favourable effects on waist circumference and sugar-sweetened beverage consumption among boys, although no effect on BMI was observed. The changes observed were consistent with the growing evidence base on whole-of-community interventions targeting childhood obesity. With the early cessation of HTV, the long-term implications of this novel government-led systems approach to prevention are unknown. The embedding of a CRT in this initiative and the use of an opt-out approach for participant recruitment seemed worthwhile approaches to evaluating the effects of the intervention on school children. Interventions delivered at this large scale, however, will likely require substantial time (e.g., 5 to 10 years) to see a substantial change. Further understanding of what makes these interventions effective would be valuable to policy makers, implementers, clinicians, and communities.

### Supplementary Information

**Additional file 1: Supplementary Table 1.** Student response rate by condition 2014 and 2016. **Supplementary Table 2.** Demographic characteristics and anthropomorphic, behavioural, quality of life, and depressive symptom outcomes by wave and trial condition for secondary school boys. **Supplementary Table 3.** Demographic characteristics and anthropomorphic, behavioural, quality of life, and depressive symptom outcomes by wave and trial condition for secondary school girls.

## Data Availability

The dataset analysed during the current study are not publicly available due the conditions under which ethics approval was granted but are available from the corresponding author on reasonable request.

## Abbreviations

BMI: Body mass index
BMIz: Body mass index z-scores
CI: Confidence interval
CRT: Cluster randomised trial
GEE: Generalised estimating equations
HTV: Healthy Together Victoria
ICSEA: Index of community socio-educational advantage
MVPA: Moderate-to-vigorous physical activity
PedsQL™ 4.0: Paediatric quality of life inventory 4.0
SEIFA: Socioeconomic index for areas
SMD: Standardised mean difference
SMFQ: Short mood and feelings questionnaire
WHOSTOPS: Whole of systems trial of prevention strategies for childhood obesity
