# Supplementary material for: Healthy together Victoria and childhood obesity study: effects of a large scale, community-based cluster randomised trial of a systems thinking approach for the prevention of childhood obesity among secondary school students 2014–2016
Source: BMC Public Health. 2024 Feb 2;24:355. doi: 10.1186/s12889-024-17906-2 (PMC10835842; doi:10.1186/s12889-024-17906-2)
Supplement: Supplementary file 1 — Additional file 1: Supplementary Table 1. Student response rate by condition 2014 and 2016. Supplementary Table 2. Demographic characteristics and anthropomorphic, behavioural, quality of life, and depressive symptom outcomes by wave and trial condition for secondary school boys. Supplementary Table 3. Demographic characteristics and anthropomorphic, behavioural, quality of life, and depressive symptom outcomes by wave and trial condition for secondary school girls. [file 12889_2024_17906_MOESM1_ESM.docx]

**Supplementary Table 1 – Student response rate by condition 2014 and 2016**

| **Intervention 2014** | | | | | | **Control 2014** | | | | | |  |
| --- | --- | --- | --- | --- | --- | --- | --- | --- | --- | --- | --- | --- |
|  | Enrolled | Consent | Absent | Opt-Out | RR |  | Enrolled | Consent | Absent | Opt-Out | RR |  |
|  |  |  |  |  |  |  |  |  |  |  |  |  |
| School I1 | 91 | 70 | 15 | 6 | 92.1 | School C1 | 397 | 232 | 69 | 96 | 70.7 |  |
| School I2 | 285 | 207 | 54 | 24 | 89.6 | School C2 | 189 | 156 | 29 | 4 | 97.5 |  |
| School I3 | 363 | 231 | 88 | 44 | 84.0 | School C3 | 94 | 55 | 20 | 19 | 74.3 |  |
| School I4 | 645 | 441 | 88 | 116 | 79.2 | School C4 | 333 | 228 | 63 | 43 | 84.4 |  |
| School I5 | 346 | 272 | 48 | 26 | 91.3 | School C5 | 166 | 99 | 50 | 17 | 85.3 |  |
| School I6 | 40 | 21 | 4 | 15 | 58.3 | School C6 | 344 | 269 | 54 | 21 | 92.8 |  |
| School I7 | 326 | 235 | 57 | 34 | 87.4 | School C7 | 133 | 94 | 25 | 14 | 87.0 |  |
| School I8 | 128 | 100 | 26 | 2 | 98.0 | School C8 | 105 | 78 | 22 | 5 | 94.0 |  |
| School I9 | 284 | 196 | 45 | 43 | 82.0 | School C9 | 23 | 18 | 1 | 4 | 81.8 |  |
| School I10 | 76 | 57 | 16 | 3 | 95.0 | School C10 | 65 | 44 | 13 | 8 | 84.6 |  |
| School I11 | 7 | 5 | 1 | 1 | 83.3 |  |  |  |  |  |  |  |
| School I12 | 158 | 128 | 25 | 5 | 96.2 |  |  |  |  |  |  |  |
| School I13 | 82 | 58 | 20 | 4 | 93.5 |  |  |  |  |  |  |  |
|  |  |  |  |  |  |  | | | | | |  |
| **Total 2014** | **2831** | **2021** | **487** | **323** | **86.2** |  | **1849** | **1273** | **346** | **231** | **84.7** |  |
|  |  |  |  |  |  |  |  |  |  |  |  |  |
| **Intervention 2016** | | | | | | **Control 2016** | | | | | |  |
|  | Enrolled | Consent | Absent | Opt-Out | RR |  | Enrolled | Consent | Absent | Opt-Out | RR |  |
| School I1 | 101 | 69 | 14 | 18 | 79.3 | School C11 | 468 | 380 | 83 | 5 | 98.7 |  |
| School I4 | 473 | 360 | 107 | 6 | 98.4 | School C2 | 206 | 135 | 53 | 18 | 88.2 |  |
| School I7 | 300 | 227 | 35 | 38 | 85.7 | School C3 | 87 | 71 | 10 | 6 | 92.2 |  |
| School I15 | 126 | 88 | 34 | 4 | 95.7 | School C12 | 71 | 60 | 7 | 4 | 93.8 |  |
| School I 16 | 360 | 216 | 78 | 20 | 76.6 | School C4 | 256 | 220 | 32 | 4 | 98.2 |  |
| School I10 | 65 | 61 | 4 | 0 | 100.0 | School C13 | 304 | 237 | 50 | 17 | 93.3 |  |
| School I11 | 10 | 8 | 2 | 0 | 100.0 | School C5 | 162 | 112 | 18 | 32 | 77.8 |  |
| School I17 | 48 | 37 | 9 | 2 | 94.9 | School C6 | 312 | 203 | 54 | 55 | 78.7 |  |
| School I18 | 65 | 53 | 9 | 3 | 94.6 | School C14 | 18 | 17 | 1 | 0 | 100.0 |  |
| School I19 | 342 | 276 | 53 | 13 | 95.5 | School C7 | 110 | 99 | 4 | 7 | 93.4 |  |
| School I20 | 396 | 302 | 58 | 36 | 89.3 | School C15 | 171 | 145 | 20 | 6 | 96.0 |  |
| School I21 | 5 | 4 | 1 | 0 | 100.0 | School C16 | 14 | 11 | 3 | 0 | 100.0 |  |
| School I22 | 252 | 184 | 45 | 23 | 88.9 | School C17 | 90 | 64 | 13 | 3 | 83.1 |  |
| School I13 | 300 | 208 | 29 | 63 | 76.8 |  |  |  |  |  |  |  |
| School I23 | 106 | 77 | 21 | 8 | 90.6 |  |  |  |  |  |  |  |
|  |  |  |  |  |  |  |  |  |  |  |  |  |
| **Total 2016** | **2949** | **2170** | **499** | **234** | **88.6** |  | **2269** | **1754** | **348** | **157** | **91.3** |  |

*Schools highlighted in green represent repeated schools in 2014 and 2016; RR = Response Rate and calculated by Consent /(Enrolled-Absent)*100

**Supplementary Table 2** Demographic characteristics and anthropomorphic, behavioural, quality of life, and depressive symptom outcomes by wave and trial condition for secondary school boys.

|  | Intervention | | | | Comparison | | | | Change between 2016 and 2014 | | | | Difference in change | |
| --- | --- | --- | --- | --- | --- | --- | --- | --- | --- | --- | --- | --- | --- | --- |
|  | 2014 | | 2016 | | 2014 | | 2016 | | Intervention | | Comparison | | Intervention vs Comparison | |
|  | N | Estimate  (95% CI) | N | Estimate  (95% CI) | N | Estimate  (95% CI) | N | Estimate  (95% CI) | Estimate  (95% CI) | P | Estimate  (95% CI) | P | Estimate  (95% CI) | P |
| **Demographic Characteristics** |  |  |  |  |  |  |  |  |  |  |  |  |  |  |
| Age (years) | 1099 | 15.1 (14.8, 15.3) | 1200 | 15.0 (14.8, 15.2) | 680 | 15.1 (14.8, 15.3) | 894 | 15.0 (14.7, 15.2) | -0.1 (-0.2, 0.1) | 0.379 | -0.1 (-0.2, 0.0) | 0.123 | 0.0 (-0.2, 0.1) | 0.615 |
| English spoken at home (%) | 1085 | 76.6 (64.1, 89.1) | 1183 | 78.9 (67.9, 89.8) | 670 | 72.1 (59.8, 84.3) | 858 | 74.5 (62.7, 86.2) | 2.4 (-3.0, 7.8) | 0.379 | 2.3 (-1.6, 6.3) | 0.245 | -0.1 (-6.7, 6.6) | 0.983 |
| **Anthropometrics** |  |  |  |  |  |  |  |  |  |  |  |  |  |  |
| BMIz (WHO) | 977 | 0.55 (0.39, 0.7) | 1053 | 0.52 (0.39, 0.66) | 643 | 0.42 (0.25, 0.58) | 770 | 0.44 (0.27, 0.6) | -0.02 (-0.18, 0.13) | 0.771 | 0.02 (-0.14, 0.18) | 0.813 | -0.04 (-0.26, 0.18) | 0.707 |
| Overweight and obese (%) | 977 | 35.5 (30.9, 40.1) | 1053 | 32.7 (28.5, 36.9) | 643 | 29.5 (25.9, 33.0) | 770 | 29.9 (25.8, 34.0) | -2.7 (-6.4, 0.9) | 0.142 | 0.4 (-3.4, 4.2) | 0.833 | -3.1 (-8.6, 2.3) | 0.259 |
| Waist circumference (cm) | 987 | 77.3 (75.7, 78.8) | 1037 | 76.6 (75.2, 78.0) | 643 | 75.3 (73.6, 77.0) | 765 | 77.1 (75.5, 78.8) | -0.7 (-2.1, 0.8) | 0.358 | 1.8 (0.4, 3.3) | 0.014 | -2.5 (-4.6, -0.5) | 0.016 |
| Abdominal obesity (%) | 987 | 11.2 (7.6, 14.7) | 1037 | 13.0 (9.4, 16.7) | 643 | 8.0 (5.4, 10.6) | 765 | 11.1 (7.6, 14.6) | 3.1 (-1.0, 7.2) | 0.138 | 1.9 (-1.0, 4.8) | 0.203 | -1.2 (-6.3, 3.8) | 0.635 |
| **Behaviour** |  |  |  |  |  |  |  |  |  |  |  |  |  |  |
| Met physical activity guideline (5 days) (%) | 1090 | 29.7 (26.8, 32.6) | 1188 | 32.1 (29.1, 35.1) | 671 | 30.0 (25.1, 34.9) | 864 | 33.1 (28.3, 37.9) | 2.3 (-0.8, 5.5) | 0.141 | 3.1 (-5.0, 11.2) | 0.456 | -0.7 (-9.2, 7.7) | 0.864 |
| Met sedentary guideline (5 days) (%) | 1085 | 52.6 (46.9, 58.3) | 1124 | 56.2 (51.8, 60.6) | 671 | 46.5 (42.0, 51.0) | 823 | 51.4 (46.5, 56.2) | 3.6 (-0.8, 8.1) | 0.110 | 4.8 (1.0, 8.6) | 0.013 | -1.2 (-7.0, 4.6) | 0.685 |
| Active transport to or from school (%) | 1009 | 37.9 (30.1, 45.8) | 1190 | 36.8 (29.1, 44.4) | 618 | 32.3 (23.3, 41.3) | 867 | 38.1 (31.8, 44.5) | -1.2 (-6.0, 3.7) | 0.638 | 5.8 (-0.1, 11.8) | 0.053 | -7.0 (-14.5, 0.5) | 0.066 |
| Met fruit guideline (%) | 1082 | 64.3 (58.1, 70.4) | 1154 | 60.9 (54.8, 67.0) | 662 | 67.7 (61.9, 73.4) | 844 | 66.2 (61.8, 70.7) | -3.3 (-10.5, 3.8) | 0.362 | -1.5 (-6.6, 3.7) | 0.583 | -1.9 (-10.6, 6.8) | 0.670 |
| Met vegetable guideline (%) | 1081 | 4.7 (2.9, 6.5) | 1185 | 6.6 (4.6, 8.5) | 660 | 4.9 (2.9, 6.9) | 862 | 7.0 (4.6, 9.5) | 1.8 (-0.3, 4.0) | 0.093 | 2.1 (-0.4, 4.6) | 0.096 | -0.3 (-3.3, 2.8) | 0.857 |
| Dairy (≥3 serves/day) (%) | 1081 | 68.1 (64.6, 71.5) | 1189 | 59.0 (55.7, 62.3) | 671 | 64.5 (61.5, 67.6) | 866 | 55.3 (50.7, 59.9) | -9.1 (-12.1, -6.0) | <0.001 | -9.2 (-14.5, -4.0) | 0.001 | 0.2 (-5.7, 6.1) | 0.954 |
| Sugar-sweetened beverages (<1/day) (%) | 1080 | 30.8 (26.0, 35.5) | 1188 | 30.7 (25.6, 35.9) | 669 | 35.5 (30.8, 40.2) | 861 | 26.9 (22.3, 31.6) | 0.0 (-6.5, 6.4) | 0.989 | -8.6 (-13.0, -4.1) | <0.001 | 8.5 (0.6, 16.5) | 0.035 |
| Packaged snacks (<1/day) (%) | 1079 | 53.8 (48.3, 59.4) | 1039 | 48.6 (43.0, 54.2) | 664 | 50.7 (43.4, 57.9) | 803 | 48.8 (42.2, 55.5) | -5.3 (-11.0, 0.4) | 0.069 | -1.8 (-8.8, 5.2) | 0.611 | -3.5 (-12.4, 5.5) | 0.447 |
| Takeaway (≤1/week) (%) | 1087 | 20.4 (16.2, 24.7) | 1190 | 20.0 (15.1, 24.9) | 670 | 19.4 (15.1, 23.8) | 867 | 18.4 (13.8, 23.1) | -0.4 (-4.1, 3.3) | 0.822 | -1.0 (-3.8, 1.9) | 0.493 | 0.6 (-4.1, 5.3) | 0.812 |
| **Health-Related Quality of Life** |  |  |  |  |  |  |  |  |  |  |  |  |  |  |
| Global score | 1080 | 81.0 (79.9, 82.2) | 1161 | 78.5 (77.5, 79.5) | 671 | 79.5 (78.2, 80.7) | 849 | 78.2 (77.0, 79.4) | -2.6 (-4.0, -1.2) | <0.001 | -1.3 (-2.7, 0.2) | 0.092 | -1.3 (-3.3, 0.7) | 0.195 |
| Physical functioning | 1083 | 87.5 (86.3, 88.7) | 1176 | 85.3 (84.3, 86.4) | 672 | 86.5 (85.2, 87.8) | 858 | 85.5 (84.3, 86.8) | -2.2 (-3.6, -0.7) | 0.003 | -1.0 (-2.4, 0.5) | 0.205 | -1.2 (-3.2, 0.8) | 0.245 |
| Psychosocial functioning | 1080 | 77.6 (76.3, 79.0) | 1162 | 74.7 (73.6, 75.9) | 673 | 75.8 (74.3, 77.2) | 851 | 74.2 (72.8, 75.6) | -2.9 (-4.5, -1.3) | 0.001 | -1.6 (-3.3, 0.1) | 0.070 | -1.3 (-3.7, 1.0) | 0.253 |
| **Depressive Symptoms** |  |  |  |  |  |  |  |  |  |  |  |  |  |  |
| Depressive symptomatology | 1053 | 3.3 (2.9, 3.7) | 1114 | 4.0 (3.6, 4.3) | 653 | 3.7 (3.3, 4.1) | 802 | 4.3 (3.9, 4.6) | 0.6 (0.2, 1.1) | 0.011 | 0.5 (0.0, 1.1) | 0.040 | 0.1 (-0.6, 0.8) | 0.807 |
| Potential depressive disorder (%) | 1053 | 11.7 (8.3, 15.1) | 1114 | 15.8 (12.8, 18.8) | 653 | 15.3 (10.6, 19.9) | 802 | 17.2 (14.6, 19.8) | 4.1 (0.7, 7.6) | 0.019 | 2.0 (-1.7, 5.6) | 0.293 | 2.2 (-2.5, 6.8) | 0.367 |

**Supplementary Table 3** Demographic characteristics and anthropomorphic, behavioural, quality of life, and depressive symptom outcomes by wave and trial condition for secondary school girls.

|  | Intervention | | | | Comparison | | | | Change between 2016 and 2014 | | | | Difference in change | |
| --- | --- | --- | --- | --- | --- | --- | --- | --- | --- | --- | --- | --- | --- | --- |
|  | 2014 | | 2016 | | 2014 | | 2016 | | Intervention | | Comparison | | Intervention vs Comparison | |
|  | N | Estimate (95% CI) | N | Estimate (95% CI) | N | Estimate (95% CI) | N | Estimate (95% CI) | Estimate (95% CI) | P | Estimate (95% CI) | P | Estimate (95% CI) | P |
| **Demographic Characteristics** |  |  |  |  |  |  |  |  |  |  |  |  |  |  |
| Age (years) | 912 | 14.9 (14.7, 15.2) | 1031 | 15.0 (14.7, 15.2) | 590 | 14.9 (14.6, 15.1) | 835 | 14.9 (14.7, 15.2) | 0.0 (-0.1, 0.2) | 0.672 | 0.1 (-0.1, 0.2) | 0.474 | 0.0 (-0.2, 0.2) | 0.837 |
| English spoken at home (%) | 900 | 76.8 (65.1, 88.5) | 1023 | 75.0 (63.0, 87.0) | 574 | 78.6 (68.6, 88.5) | 815 | 78.4 (67.5, 89.4) | -0.2 (-5.0, 4.7) | 0.951 | -1.8 (-4.0, 0.4) | 0.112 | -1.6 (-6.9, 3.6) | 0.542 |
| **Anthropometrics** |  |  |  |  |  |  |  |  |  |  |  |  |  |  |
| BMIz (WHO) | 764 | 0.55 (0.41, 0.68) | 887 | 0.62 (0.5, 0.73) | 496 | 0.51 (0.37, 0.65) | 624 | 0.67 (0.54, 0.81) | 0.07 (-0.07, 0.22) | 0.333 | 0.16 (0.01, 0.31) | 0.032 | -0.09 (-0.3, 0.12) | 0.395 |
| Overweight and obese (%) | 764 | 31.9 (27.3, 36.6) | 887 | 35.7 (30.0, 41.3) | 496 | 30.4 (25.2, 35.5) | 624 | 35.4 (30.1, 40.7) | 3.8 (-2.1, 9.6) | 0.209 | 5.0 (-0.3, 10.3) | 0.063 | -1.3 (-9.3, 6.8) | 0.759 |
| Waist circumference (cm) | 795 | 73.6 (72.2, 75.0) | 876 | 73.8 (72.5, 75.0) | 509 | 73.2 (71.7, 74.7) | 626 | 74.5 (73.1, 76.0) | 0.2 (-1.2, 1.6) | 0.794 | 1.3 (0.0, 2.7) | 0.058 | -1.1 (-3.1, 0.8) | 0.245 |
| Abdominal obesity (%) | 795 | 22.5 (16.3, 28.7) | 876 | 23.5 (16.5, 30.4) | 509 | 19.4 (12.1, 26.7) | 626 | 22.2 (16.8, 27.6) | 1.0 (-6.4, 8.4) | 0.789 | 2.8 (-3.5, 9.1) | 0.387 | -1.8 (-12.0, 8.5) | 0.734 |
| **Behaviour** |  |  |  |  |  |  |  |  |  |  |  |  |  |  |
| Met physical activity guideline (5 days) (%) | 898 | 14.3 (11.0, 17.7) | 1029 | 19.3 (15.6, 22.9) | 584 | 15.2 (11.9, 18.6) | 818 | 17.8 (14.0, 21.7) | 4.9 (2.2, 7.7) | <0.001 | 2.6 (-0.8, 6.0) | 0.134 | 2.3 (-1.9, 6.5) | 0.277 |
| Met sedentary guideline (5 days) (%) | 897 | 53.7 (46.1, 61.2) | 981 | 56.1 (49.6, 62.7) | 582 | 47.7 (40.6, 54.8) | 776 | 54.7 (49.6, 59.8) | 2.5 (-5.4, 10.4) | 0.541 | 7.0 (0.9, 13.0) | 0.023 | -4.5 (-14.2, 5.2) | 0.361 |
| Active transport to or from school (%) | 839 | 30.6 (23.8, 37.4) | 1027 | 33.4 (27.4, 39.5) | 556 | 29.7 (23.2, 36.2) | 818 | 30.5 (25.4, 35.6) | 2.8 (-2.1, 7.8) | 0.258 | 0.8 (-4.3, 6.0) | 0.755 | 2.0 (-4.8, 8.8) | 0.562 |
| Met fruit guideline (%) | 901 | 71.9 (65.3, 78.5) | 994 | 67.1 (62.0, 72.2) | 582 | 72.5 (67.3, 77.7) | 796 | 67.8 (63.7, 71.8) | -4.8 (-9.5, -0.1) | 0.046 | -4.7 (-9.0, -0.4) | 0.030 | -0.1 (-6.3, 6.1) | 0.982 |
| Met vegetable guideline (%) | 900 | 8.6 (6.5, 10.8) | 1026 | 13.1 (10.9, 15.4) | 578 | 5.4 (3.5, 7.3) | 817 | 12.5 (10.6, 14.5) | 4.5 (1.6, 7.4) | 0.002 | 7.2 (5.1, 9.3) | <0.001 | -2.7 (-6.1, 0.7) | 0.122 |
| Dairy (≥3 serves/day) (%) | 893 | 77.7 (73.8, 81.5) | 1025 | 69.8 (66.5, 73.1) | 580 | 75.6 (71.8, 79.3) | 815 | 74.5 (70.1, 78.9) | -7.8 (-12.8, -2.9) | 0.002 | -1.1 (-5.5, 3.4) | 0.641 | -6.8 (-13.6, 0.0) | 0.052 |
| Sugar-sweetened beverages (<1/day) (%) | 898 | 18.0 (14.8, 21.2) | 1026 | 18.7 (14.6, 22.7) | 584 | 19.1 (14.9, 23.2) | 815 | 17.1 (13.2, 21.1) | 0.7 (-3.4, 4.7) | 0.744 | -1.9 (-7.6, 3.8) | 0.504 | 2.6 (-4.4, 9.6) | 0.465 |
| Packaged snacks (<1/day) (%) | 888 | 56.7 (52.6, 60.8) | 911 | 55.7 (51.8, 59.6) | 582 | 53.4 (48.1, 58.8) | 747 | 55.1 (51.2, 58.9) | -1.0 (-6.0, 3.9) | 0.689 | 1.6 (-3.4, 6.6) | 0.525 | -2.6 (-9.6, 4.3) | 0.459 |
| Takeaway (≤1/week) (%) | 893 | 13.1 (10.1, 16.1) | 1027 | 10.6 (8.0, 13.2) | 574 | 11.6 (8.7, 14.6) | 817 | 12.6 (8.8, 16.3) | -2.5 (-5.1, 0.1) | 0.059 | 0.9 (-2.5, 4.3) | 0.593 | -3.4 (-8.0, 1.1) | 0.135 |
| **Health-Related Quality of Life** |  |  |  |  |  |  |  |  |  |  |  |  |  |  |
| Global score | 896 | 75.0 (73.5, 76.6) | 1010 | 73.3 (71.9, 74.6) | 585 | 73.9 (72.2, 75.5) | 802 | 73.5 (72.0, 75.0) | -1.8 (-3.5, -0.1) | 0.037 | -0.4 (-2.1, 1.3) | 0.665 | -1.4 (-3.8, 0.9) | 0.239 |
| Physical functioning | 897 | 82.0 (80.5, 83.4) | 1020 | 80.6 (79.4, 81.9) | 585 | 80.9 (79.3, 82.5) | 812 | 81.5 (80.1, 83.0) | -1.3 (-3.0, 0.3) | 0.119 | 0.6 (-1.1, 2.3) | 0.476 | -1.9 (-4.3, 0.4) | 0.105 |
| Psychosocial functioning | 896 | 71.4 (69.5, 73.2) | 1010 | 69.3 (67.7, 70.8) | 585 | 70.1 (68.2, 72.1) | 803 | 69.3 (67.5, 71.1) | -2.1 (-4.1, -0.1) | 0.037 | -0.8 (-2.8, 1.1) | 0.407 | -1.3 (-4.0, 1.5) | 0.370 |
| **Depressive Symptoms** |  |  |  |  |  |  |  |  |  |  |  |  |  |  |
| Depressive symptomatology | 864 | 6.1 (5.4, 6.8) | 957 | 6.9 (6.3, 7.5) | 567 | 6.6 (5.8, 7.3) | 777 | 7.2 (6.5, 7.9) | 0.8 (0.0, 1.6) | 0.037 | 0.6 (-0.2, 1.4) | 0.117 | 0.2 (-0.9, 1.3) | 0.704 |
| Potential depressive disorder (%) | 864 | 26.5 (22.2, 30.8) | 957 | 32.7 (28.8, 36.6) | 567 | 30.8 (26.2, 35.4) | 777 | 33.6 (30.6, 36.7) | 6.1 (2.6, 9.7) | 0.001 | 2.8 (-2.2, 7.8) | 0.271 | 3.3 (-2.8, 9.5) | 0.292 |
